# An Analytical Model for Hysteretic Pressure-Sensitive Permeability of Nanoporous Media

**DOI:** 10.3390/nano12234234

**Published:** 2022-11-28

**Authors:** Gang Lei, Qinzhuo Liao, Weiqing Chen, Chunhua Lu, Xianmin Zhou

**Affiliations:** 1 Faculty of Engineering, China University of Geosciences, Wuhan 430074, China; 2 State Key Laboratory of Petroleum Resources and Prospecting, China University of Petroleum-Beijing, Beijing 102249, China; 3 College of Petroleum Engineering and Geosciences, King Fahd University of Petroleum & Minerals, Dhahran 31261, Saudi Arabia

**Keywords:** nanoporous media, hysteretic pressure-sensitive permeability, analytical model, nanoscale effects

## Abstract

Hysteretic pressure-sensitive permeability of nanohybrids composed of substantial nanopores is critical to characterizing fluid flow through nanoporous media. Due to the nanoscale effect (gas slippage), complex and heterogeneous pore structures of nanoporous media, the essential controls on permeability hysteresis of nanohybrids are not determined. In this study, a hysteretic pressure sensitive permeability model for nitrogen flow through dry nanoporous media is proposed. The derived model takes into account the nanoscale effect and pore deformation due to effective stress. The model is validated by comparing it with the experimental data. The results show that the calculated permeability and porosity are consistent with the measured results with the maximum relative error of 6.08% and 0.5%, respectively. Moreover, the hysteretic pressure-sensitive permeability of nanohybrids is related to effective stress, gas slippage, pore microstructure parameters, grain quadrilateral angle, and the loss rate of grain quadrilateral angle. The nanoscale effect is crucial to the permeability of nanoporous media. In addition, as impacted by the comprehensive impact of multiple relevant influential parameters, permeability during the pressure unloading process is not a monotonous function but presents complicated shapes. The proposed model can explain, quantify, and predict the permeability hysteresis effect of nanoporous media reasonably well.

## 1. Introduction

In recent times, to safeguard energy supply and energy security, the extraction of hydrocarbons from nanoporous media (e.g., tight sandstone and shale) containing nanoscale dominating pores has increased significantly [1,2,3,4]. In this situation, a reasonable description of fluid flow and transport through nanoporous materials (nanohybrids) is of significance in the environment and energy fields [5,6,7]. Nanohybrids (shale or mudrock) have minute pore sizes (predominantly nanoscale pores < 100 nm), which presents a strong nanoscale effect (gas slippage). Technically speaking, as the main part of the nanohybrids, the nanoscale dominating pores and throats will lead to evident nanoscale effect and nonlinear seepage characteristics [1]. Therefore, researches on the fluid flow and transport behavior in nanoporous materials (nanohybrids) are important and difficult tasks for the production of hydrocarbons.

Physically speaking, permeability is one of the basic fundamental parameters in flow equations (e.g., Darcy flow equation, Brinkman equation, and Forchheimei equation, etc.) for characterizing fluid flow and transport behaviors in porous materials [8,9,10,11,12,13,14]. It is not possible to model fluid flow behavior in porous media without having an accurate permeability value. In a nanohybrid substantially composed of nano-pores, permeability can be used for the derivation of the constitutive relations [2], hydrocarbon resource assessment [15,16,17], and well production prediction [7,18,19,20]. Gholami et al. [21] stated that permeability was the most important rock parameter affecting fluid transport through porous materials. Zhang et al. [22] suggested that modeling fluid transport behavior in nanohybrid was extremely important for the evaluation of tight reservoir performance. Consequently, a deep understanding of the permeability of nanoporous media is crucial for the optimization of tight reservoir development.

As stated in the previous research, the permeability of porous materials was sensitive to pressure (or effective stress), which displayed strong hysteresis effects [23,24,25,26,27,28,29,30,31,32,33,34,35]. For example, Farquhar et al. [24] measured the stress-dependent permeability of tight sandstones and concluded that rock permeability and pore structure parameters changed with increasing effective stress. Furthermore, they suggested that pore structure parameters reflected by rock permeability measured under low confining pressure conditions could not represent that which existed in situ. Bustin [25] carried out permeability tests on coals and found permeability changed markedly with effective stress. Based on the test data, Xiao et al. [31] suggested that the stress sensitivity of samples with the development of the crack-like pore was strong. Moreover, they concluded that the stress-dependent permeability of cores would be affected by rock grain size, rock lithology, and the types of cemented clay minerals. Geng et al. [32] conducted permeability tests on reconstituted coals under stress conditions, and they found permeability of reconstituted coal decreased exponentially as the effective stress increased. Recently, Lei et al. [16] studied the permeability of argillaceous nanoporous media under stress dependence with clay swelling. They suggested that, due to effective stress, the permeability of argillaceous nanoporous media would decrease sharply. It is common knowledge that the permeability pressure hysteresis effect is commonly encountered in hydrologic science and engineering, which occurs during the deformation of reservoir rocks. This term has been widely used in various previous studies. Physically speaking, reservoir permeability continuously decreases as effective stress increases, caused by the reduction of formation pressure. Then, to replenish and maintain the formation pressure and reduce effective stress, scientists and engineers usually inject fluid (water or gas) into the reservoir to constantly recover reservoir energy. However, during the process of injection of water and gas, reservoir permeability cannot fully recover to the initial state (or original state). This hysteresis phenomenon in porous media is known as permeability hysteresis in rock mechanics engineering. In general, permeability hysteresis is one of the main reasons for permanent permeability damage of reservoir rocks, which affects the fluid transport and flow mechanisms in porous media [30,33,36,37]. Teklu et al. [38,39] suggested that the influence of permeability hysteresis on hydrocarbon reservoir production strategies was significant and could not be ignored. Moreover, Cao and Lei [33] conducted experimental tests on tight intact cores and found a strong permeability pressure hysteresis effect in these samples. In general, it is more apparent to incur the permeability pressure hysteresis of nanoporous media with lower permeability or narrower pore radius. Chen et al. [37] studied the permeability of shale samples with abundant nanoscale pores and found nanohybrid permeability presented considerable hysteresis. Consequently, for nanoporous materials, the effect of permeability hysteresis on subsurface flow cannot be ignored, and it is of practical significance to investigate the permeability hysteresis of these nanoporous media.

It is well known that the accurate estimation of hysteretic pressure-sensitive permeability in nanohybrids is a difficult task [21]. As a basic, straightforward, and efficient way to draw causal conclusions, many scholars have carried out experimental tests (e.g., [30,33,38,39,40,41,42]) to study the permeability hysteresis of nanoporous media (such as sandstone, carbonate, shale, etc.) during pressure loading and unloading processes. Results from the tests suggest that permeability hysteresis is significantly affected by the stress path [40,41,43]. Bernabe [43] conducted stress sensitivity tests and found the hysteresis effect of rock. He also suggested that, after aging treatment, the permeability hysteresis effect would be eliminated or minimized. Teufel et al. [44], Warpinski, and Teufel [45] also found that, after multiple aging treatments, the sensitivity hysteresis effect of porous media would disappear. Ruan and Wang [46] suggested that, for nanoporous media, the increased stress would lead to elastoplastic deformation. However, during the unloading pressure process, the plastic deformation can never restore to the initial state. Wang [47] concluded that the restoration extent of nanoporous media was affected by the water injection time and initial permeability. He suggested that the stronger permeability hysteresis corresponded to the porous media with lower permeability. Teklu et al. [48] concluded that permeability hysteresis of nanohybrids was the function of pore structure and mineral composition of nanoporous media. Shi and Sun [49] suggested that compared to lithic sandstone and mudstone, quartz sandstone had a lower permeability hysteresis effect. However, although the experiment has the advantage of being repeatable, the ability of this method is sometimes limited. For example, permeability is generally measured on the cored samples taken from reservoirs. Nevertheless, the experimental tests are usually time-consuming and expensive, core data are available only for a few wells, and the experimental samples usually represent only a very small proportion of the reservoir. In addition, the experimental results are mainly qualitative works, which are highly vulnerable to extraneous variables during the tests. Moreover, the experimental results with different experimental methods had many inconsistencies due to the discrepancies in the rock’s physical properties.

To quantitatively analyze the permeability hysteresis effect of nanoporous materials, the Pore-network model, Lattice Boltzmann method, Monte Carlo method, molecular dynamics simulations, etc., have also been developed by Jerauld and Salter [50], Sarkisov and Monson [51], and Wang et al. [52]. However, as these numerical methods need accurate porous structure models of nanohybrids, the computational cost of these numerical models is extremely expensive. Furthermore, numerical simulations are sometimes not robust, and the predicted results from the numerical methods are subject to numerical dispersion. Hence, to some extent, the applicability of these numerical methods to model permeability hysteresis of nanoporous materials is sometimes limited. Due to the complex deformation mechanism, the relevant research on permeability hysteresis based on analytical modeling is scarce. In our previous study [33], an analytical model was firstly derived to discuss the permeability hysteresis effect of nanoporous media samples. However, this model is limited by assuming that the pore radius in the nanoporous media is evenly distributed, which is suspected of oversimplification and may not be reasonable for real nanohybrids. Additionally, this model ignores the nanoscale effect (gas slippage) on fluid flow through nanohybrids. Therefore, to make the model more rational, further research is needed.

Up to the present, the main mechanisms of the permeability hysteresis effect are still not definitive. Moreover, a comprehensive investigation of permeability hysteresis of nanoporous media remains elusive. Motivated by this status, this work intends to make more progress to fill this knowledge gap. Specifically, an analytical model has been proposed in this study to understand the fundamental controls on the permeability hysteresis effect of nanohybrids. Since the fractal geometry theory was introduced by Mandelbrot [53], numerous scholars [4,54,55,56,57] have suggested the interspaces in most of the nanoporous media have the fractal characteristics. Furthermore, the fractal-based models have been proven to be effective for various applications in predicting the permeability of nanoporous media [16,58]. In this study, a new hysteretic pressure-sensitive permeability analytical model of nanoporous media is developed to investigate permeability hysteresis behavior using fractal modeling. This model takes the pore size distribution and nanoscale effect (gas slippage) of nanoporous media into account. A concise outline of this paper is as follows. Firstly, experimental data of hysteretic pressure-sensitive permeability are studied, then the model of hysteretic pressure-sensitive permeability of nanoporous media is established to take account of the pore size distribution and nanoscale effect (gas slippage). Subsequently, this newly derived model is validated using the experiment results. Finally, the influences of relevant parameters on the permeability hysteresis effect are evaluated.

## 2. Materials and Methods

### 2.1. Experiment Materials and Methods

In this study, six intact tight sandstone samples whose microfractures can be ignored were prepared to conduct experiments. The main materials for making these nanoporous media were quartz sand (as the aggregates), epoxy resin, and 593 epoxy resin curing agent (as the binder). Based on the epoxy resin pressing cementation method, these samples were prepared for the permeability experiments. Specifically, these porous materials were composed of quartz sand aggregates (approximately 59.56%), the binder agent (approximately 31.81%), and pore (approximately 8.63%). Generally speaking, the bulk modulus and shear modulus of quartz sand are 37 GPa and 23.05 GPa, respectively. For the epoxy resin and 593 epoxy resin curing agent, the bulk modulus and shear modulus are 2.78 GPa and 0.72 GPa, respectively. For the pore in the porous materials, the bulk modulus is 4.07 GPa and the shear modulus is 0 GPa, which are identical to those assigned in the literature [59]. Thus, based on the Voigt–Reuss–Hill model [59,60], the effective bulk modulus and shear modulus of these samples could be determined as 23.27 GPa and 13.96 GPa, respectively. Moreover, by combining the predicted average effective bulk modulus, average effective shear modulus and the theory of mechanics of materials, the elastic modulus and Poisson’s ratio can be approximately determined as 34.9 GPa and 0.25. The diameter and length of these core samples vary between 24.7–25.2 mm (average diameter 24.9 mm) and 43.8–56.2 mm (average length 50.3 mm), respectively. The initial permeability $K_{0}$ (*K* means permeability, μ$m^{2}$; subscript “0” means initial value/state) of these samples ranges within $0.02\times 10^{-3}$–$0.15\times 10^{-3}$μ$m^{2}$ with an average permeability of $0.73\times 10^{-3}$μ$m^{2}$. In addition, the initial porosity $\varphi_{0}$ (φ means porosity, dimensionless) of these nanohybrids ranges within 4.39–12.50%. Based on the Kozeny-Carman equation $K_{0}=\varphi_{0}r_{\mathrm{av}}^{2}/8$, the average pore radius $r_{\mathrm{av}}$ (*r* means pore radius, μm/micron; subscript “av” means average) of these samples can be determined by $r_{\mathrm{av}}=\sqrt{8K_{0}/\varphi_{0}}$. Therefore, $r_{\mathrm{av}}$ ranges within 0.06037–0.09798 μm. Thus, nanoscale pores are developed in these 6 tight sandstone samples. The petrophysical properties of the core samples are summarized in Table 1. The experimental approach for hysteretic pressure-sensitive permeability in this study (e.g., experimental fluid, experimental apparatus, and experimental procedure) is identical to that published in our previous study [33]. Moreover, the experimental data regarding porosity hysteresis of nanohybrids will further validate the derived model in this work.

**Figure 1:**
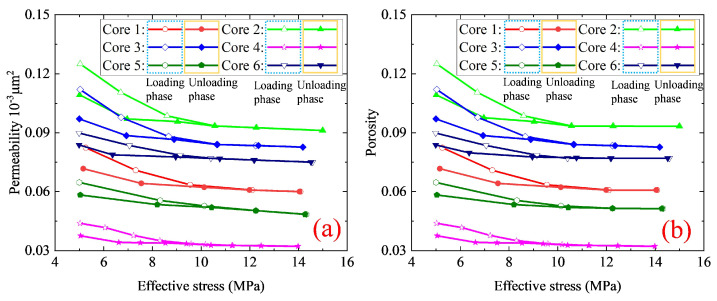
The experimental results: (**a**) the normalized permeability. (**b**) the stress-dependent porosity. The open and filled symbols in (**a**,**b**) correspond to loading and unloading phases, respectively.

The experimental procedures are identical to those stated in our previous work [33]. The experimental procedures are composed of washing and drying core samples (about 48 h), establishing experimental conditions (temperature and pressure), and executing permeability tests during pressure loading/unloading processes. The permeability hysteresis of nanohybrids can be studied from the measurements. Figure 1a,b present the normalized permeability (i.e., the ratio of stress-dependent permeability *K* to the initial permeability $K_{0}$) curves and the stress-dependent porosity curve. As illustrated in Figure 1a,b, permeability and porosity continually decrease as effective stress increases. Pore compression (e.g., permeability and porosity decreasing) during the effective stress increasing process can be represented by a two-stage process that includes the early stage and the late stage of change. Permeability or porosity decreases sharply as effective stress increases at the early stage. The change of permeability or porosity becomes weak and gradually tends to be stable at the late stage. The reason is that, during early effective stress increasing, solid material deformation and structural deformation work together, leading to a sharp decrease in permeability. When the value in effective stress increases up to a certain extent, the decrease in permeability tends to be slowed down (i.e., the structural deformation of nanoporous media will tend to be stable), and the solid material deformation of nanohybrids continues to grow [33]. Furthermore, the nanohybrid has a strong permeability hysteresis effect, which is stronger than the porosity hysteresis effect. For these six nanohybrids, the permeability ratio after the stress returning to the original state to $K_{0}$ ranges from 0.77 to 0.87. However, the porosity ratio after the stress returning to the original state ranges from 0.85 to 0.93. In other words, normalized permeability loss (the difference between unity and normalized permeability of nanoporous media after the stress returning to the original state) varies between 0.13–0.23. However, porosity loss (the difference between unity and porosity of nanoporous media after the stress returning to the original state) varies between 0.07–0.15. From our previous experiments and simulations [14,33,34] and relevant literature reviewed in the introduction section, nanoporous media displays a strong permeability hysteresis effect, which is significantly influenced by multiple influential parameters (e.g., pore structure parameters and lithology of porous materials). In the next section, an analytical model for permeability hysteresis will be proposed to incorporate the pore structure parameters and lithology of nanoporous media. Moreover, the derived model will be further validated with the experiment data.

### 2.2. Theoretical Model Development

#### 2.2.1. Model Assumptions

**Figure 2:**
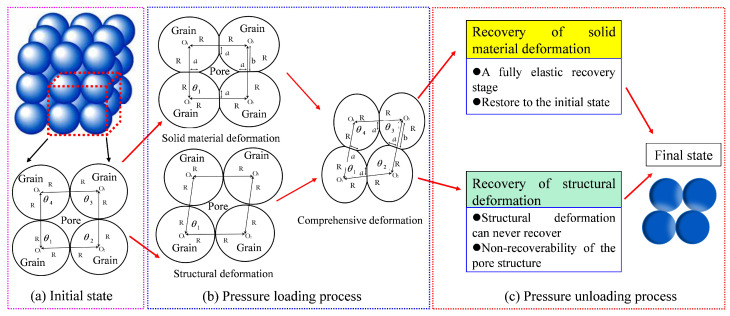
Pore deformation during pressure loading and unloading processes: (**a**) Initial state. (**b**) Pressure loading process. (**c**) Pressure unloading process.

For nitrogen flow through nanohybrid driven by the differential pressure, it is assumed that the nanohybrid is subjected to confining pressure. Due to the effective stress (i.e., the difference between the confining pressure exerted on the porous materials and the pore pressure of fluid existing in the pore space of porous materials), the nanohybrid will be compressed and its permeability will obviously change, leading to the change of nitrogen flow in the nanoporous materials. In general, with the increase of effective stress, fully elastic deformation, elastoplastic deformation, and fully plastic deformation will occur in sequence [61]. However, as elastoplastic deformation and fully plastic deformation are extremely complex, for the sake of simplification, in this paper, we focus on the fully elastic deformation of nanohybrids. In other words, the effective stress in this paper is smaller than the rock yield stress, and we consider the fully elastic deformation of rocks. It is supposed that each pore in the nanohybrid is a void space between 4 identical spherical grains (e.g., the grain size, the elastic modulus, and Poisson’s ratio) arranged in a specific packing (shown in Figure 2a). During the pressure loading process, spherical grains will be deformed, and the corresponding intergrain pore will be compressed (e.g., both pore shape and pore size vary with the pressure). However, during the pressure unloading process, the intergrain pore radius (i.e., the equivalent pore radius of the void space between 4 identical spherical grains) can never recover. The analytical permeability hysteresis model is deduced under the following assumptions:

(1) During fluid flow through nanohybrids, the fluid velocity is assumed to be approximately symmetrically distributed with respect to the tube axis. In addition, as pores in nanohybrids are narrow, due to the gas slippage effect, the fluid velocity at the pore wall is larger than zero.
(2) The effective stress is uniformly distributed in the pore space of the porous materials during pressure loading and unloading processes and act in the same way on all pores in the material. The increased effective stress leads to solid material deformation and structural deformation, as shown in Figure 2b. During the solid material (grains themselves) deformation process, the point contacts between grains transform into surface contacts, determined by the Hertz deformation theory. In other words, solid material deformation is fully elastic deformation. Moreover, during the structural deformation process, particle contact angle changes as the arrangement of grains changes.
(3) When the effective stress decreases (i.e., pressure unloading), the nanoporous media’s solid material deformation will revert to the initial state. This process is a fully elastic recovery stage, as shown in Figure 2c. However, the nanoporous media’s structural deformation can never recover, leading to the non-recoverability of the pore structure.
(4) During the process of effective stress increasing or decreasing, rock mechanical properties (e.g., elastic modulus and Poisson’s ratio) are considered constants. In other words, the variations in the mechanical properties of the nanohybrid are ignored.
(5) The samples are dry nanoporous media and the liquid film on the pore wall is ignored. In addition, as the fluid is nitrogen gas, the adsorption of nitrogen on nanopore’s surface is also ignored. The flow in a circular intergrain nanopore is laminar and in a steady state, and the velocity distribution in the nanopore can be characterized by the modified Navier-Stokes equation [22,62,63,64,65].

#### 2.2.2. Theoretical Models

According to Hertz’s deformation theory, due to the exerting force *F*, the contact radius of nano-particles *a* can be expressed as the following equation [26,33,61,66]:

(1) $$a=\sqrt[{3}]{\frac{3F}{4}\frac{R_{1}R_{2}}{R_{1}+R_{2}}\left(\frac{1-\nu_{1}^{2}}{E_{1}}+\frac{1-\nu_{2}^{2}}{E_{2}}\right)}=\sqrt[{3}]{\frac{3F}{4}\frac{R\left(1-\nu^{2}\right)}{E}},$$

where *a* means contact radius (μm), *F* means force (N), *R* denotes the equivalent spherical particle radius (μm), which is identical to $R_{1}R_{2}/\left(R_{1}+R_{2}\right)$. The subscript of “1” means particle 1. The subscript “2” means particle 2. Supposed that $R_{1}$ is identical to $R_{2}$, the equivalent radii *R* can be simplified as R/2. *E* represents the equivalent elastic modulus (GPa), which is determined by $E={\left(\frac{1-\nu_{1}^{2}}{E_{1}}+\frac{1-\nu_{2}^{2}}{E_{2}}\right)}^{-1}$, where ν means the Poisson ratio (dimensionless). It is general knowledge that Equation (1) is derived according to the assumption that the particle surface is sufficiently smooth and the particle is completely elastic, which neglects the surface topological features of the natural rock grains and mineral composition. So, for typical rough particle surfaces, Equation (1) can be modified as [57]:

(2) $$\left\{\begin{array}{c} a={\left[\frac{3F}{4}\frac{R\left(1-\nu^{2}\right)}{E}\right]}^{\omega }\\ b=\sqrt{R^{2}-a^{2}} \end{array}\right.,$$

where *b* is the distance from the grain center to the contact surface (μm), ω denotes a parameter representing the rough particle surfaces (dimensionless).

In Equation (2), if parameter ω is assigned as 1/3, the equation is in line with the classical Hertz deformation theory. However, ω should be larger than 1/3 when particle surfaces are rough. It reveals that, compared with the classical Hertz deformation theory, Equation (2) is more general. In other words, the classical Hertz deformation theory can be regarded as a special case of Equation (2).

Based on the definition of effective stress, we have [61]:

(3) $$\sigma_{\mathrm{jp}}=\frac{F}{\pi a^{2}}+\sigma_{0}=\frac{F}{\pi }{\left[\frac{3F}{4}\frac{R\left(1-\nu^{2}\right)}{E}\right]}^{-2\omega }+\sigma_{0},$$

where parameter σ is the effective stress (MPa), subscript “jp” means the value in the pressure loading process, parameter $\sigma_{0}$ is the initial effective stress (MPa). If we assume $\sigma_{0}$ is equal to 0 MPa, then effective stress $\sigma_{\mathrm{jp}}=F/\left(\pi a^{2}\right)$ is identical to that defined by [61]. With the determined effective stress during the effective stress increasing process using Equation (3), the porous radius *r* is [67]:

(4) $$r=R\sqrt{\frac{4b^{2}}{\pi R^{2}}\sin\theta_{1}-\frac{4ab}{\pi R^{2}}-\left(1-\frac{4}{\pi }\arctan\frac{a}{b}\right)},$$

where parameter $\theta_{1}$ denotes the interior angle (the contact angle, seen in Figure 2) of the quadrilateral formed by the center points of four spherical particles during the pressure loading process (rad). On the whole, the packing models of particles for actual porous materials are extremely complex and difficult to be characterized. As the initial state refers to the state where effective stress is zero and particle change never occur, to simplify the model, many scholars [66,68,69,70] assumed that pores of porous materials under zero effective stress could be represented as the packing of particles with the identical size. Specifically, Weaver et al. [68,69], and Terracina et al. [70] suggested that pores of porous materials could be represented by the cubic packing of 4 particles with identical size. In this paper, for the sake of simplicity, the cubic packing model of 4 spherical particles is applied to characterize the pore structure of porous materials under zero effective stress (i.e., the initial state, seen in Figure 2a). Thus, it is reasonable to assume that, under the initial state, the contact radius *a* is equal to 0 μm, and parameters *b* and $\theta_{1}$ are equal to particle radius *R* and π/2, respectively. Mathematically speaking, the initial pore radius of nanoporous media $r_{0}$ can be determined by simplifying Equation (4) as $r_{0}=R\sqrt{(4-\pi )/\pi }$.

By substituting $r_{0}$ into Equation (4), the pore radius *r* is

(5) $$r=r_{0}\frac{\sqrt{\frac{4b^{2}}{\pi R^{2}}\sin\theta_{1}-\frac{4ab}{\pi R^{2}}-\left(1-\frac{4}{\pi }\arctan\frac{a}{b}\right)}}{\sqrt{(4-\pi )/\pi }},$$

in which the stress-dependent contact angle $\theta_{1}$ changes in the range of $\pi /3\leq \theta_{1}$≤π/2, which reflects the structural deformation of nanohybrids. Mathematically, we have $\frac{\sqrt{\frac{4b^{2}}{\pi R^{2}}\sin\theta_{1}-\frac{4ab}{\pi R^{2}}-\left(1-\frac{4}{\pi }\arctan\frac{a}{b}\right)}}{\sqrt{(4-\pi )/\pi }}\leq 1$. So, the pore radius will decrease after deformation, which is expected. Equation (5) demonstrates that *r* depends on parameter $\theta_{1}$, which ranges from π/3-π/2. Physically speaking, contact angle $\theta_{1}$ is related to pore structure (pore geometry) and effective stress. For example, Equation (5) reveals that parameter $\theta_{1}$ decreases monotonically with the increase of effective stress. In this paper, for the sake of simplification, an equation describing the change of $\theta_{1}$ given by [33] is as follows:

(6) $$\theta_{1}=\left\{\begin{array}{c} \frac{\pi }{3}+\frac{\pi }{6}{\left(\frac{\sigma_{0}}{\sigma_{\mathrm{jp}}}\right)}^{\frac{\sigma_{\mathrm{jp}}\cdot \beta }{\sigma_{0}\cdot \Pi }},\;\frac{\sigma_{\mathrm{jp}}}{\sigma_{0}}\leq \Pi \\ \frac{\pi }{3}+\frac{\pi }{6}\cdot \frac{1}{\Pi^{\beta }},\;\;\;\frac{\sigma_{\mathrm{jp}}}{\sigma_{0}}>\Pi \end{array}\right.,$$

where parameter β presents the changing rate of the contact angle $\theta_{1}$ hereinafter (dimensionless), and Π denotes the parameter representing the effective stress when nanohybrid structural deformation stops varying (dimensionless). Cao et al. [33] suggested that parameter Π could be assigned 2 in the calculation. However, due to the complex pore structure, mineral composition, and lithology, parameter Π for different nanohybrids will vary. For example, due to the influence of many factors such as the geological deposit and evolution, parameter Π for actual nanohybrids could not always be 2. The accurate value of parameter Π needs to be obtained from experimental tests.

Based on Equations (1) and (4), the following equation for the maximum and minimum pore radius is

(7) $$\left\{\begin{array}{c} r_{\max,\;0}=R_{\max}\sqrt{(4-\pi )/\pi }\\ r_{\min,\;0}=R_{\min}\sqrt{(4-\pi )/\pi }\\ r_{\max}=R_{\max}\sqrt{\frac{4b_{\max}^{2}}{\pi R_{\max}^{2}}\sin\theta_{1}-\frac{4a_{\max}b_{\max}}{\pi R_{\max}^{2}}-\left(1-\frac{4}{\pi }\arctan\frac{a_{\max}}{b_{\max}}\right)}\\ r_{\min}=R_{\min}\sqrt{\frac{4b_{\min}^{2}}{\pi R_{\min}^{2}}\sin\theta_{1}-\frac{4a_{\min}b_{\min}}{\pi R_{\min}^{2}}-\left(1-\frac{4}{\pi }\arctan\frac{a_{\min}}{b_{\min}}\right)} \end{array}\right.,$$

where subscript “max” means maximum value, subscript “min” means minimum value, *R*_max_ means the maximum equivalent spherical particle radius, *R*_min_ means the minimum equivalent spherical particle radius. In general, the nanohybrid contains grains sizes of various sizes and pores may be framed by the packing of particles with different dimen-sions. However, as mentioned in the Introduction, due to the complexity of the random and disordered pore structure of nanohybrid, it is extremely difficult to quantify the pore structure of nanohybrid. In this paper, to simplify the model, the polydispersity of particle packing and the pores constituted between particles/grains with different dimensions are ignored, and it is assumed that the pores are constituted by the packing of particles with the identical dimension and physical properties. Thus, the maximum and minimum pore radius correspond to the packing of particles with the maximum and minimum particle dimensions, respectively. In addition, it should be noted that, as the polydispersity of particle packing and the pores constituted between particles/grains with different dimensions are ignored, and the effective stress is uniformly distributed in the pore space, it is reasonable to assume $\theta_{1}$ in Equation (7) is the same for larger grains and smaller ones.

It should be noted that parameter *R* is not the actual size of nano-particles but the equivalent particle radius, which accounts for mineral composition in nanohybrids. In general, there are clay minerals, cementing materials, and other minerals in pore space for actual nanohybrids, which will narrow pore size. Based on fractal theory and nitrogen flow through nanohybrids, the permeability of nanohybrids can be determined as Equation (A10). More details of Equation (A10) can be found in Appendix A. By combining Equations (7) and (A10), the initial permeability $K_{0}$ for the dry nanohybrids is

(8) $$K_{0}=\frac{2^{D_{T0}}\times \pi \times D_{f0}\times r_{\max,\;0}^{D_{f0}-D_{T0}-1}}{16\times {\left(\frac{\pi \times D_{f0}}{2-D_{f0}}\times \frac{1-\varphi_{0}}{\varphi_{0}}\right)}^{\frac{D_{T0}+1}{2}}}\left(\begin{array}{c} \frac{r_{\max,\;0}^{3+D_{T0}-D_{f0}}-r_{\min,\;0}^{3+D_{T0}-D_{f0}}}{3+D_{T0}-D_{f0}}+\\ 4\frac{2-\sigma_{v}}{\sigma_{v}}\frac{\xi }{1-b_{0}\xi }\frac{r_{\max,\;0}^{2+D_{T0}-D_{f0}}-r_{\min,\;0}^{2+D_{T0}-D_{f0}}}{2+D_{T0}-D_{f0}} \end{array}\right),$$

wherein,

(9) $$\left\{\begin{array}{c} D_{f0}=2-\frac{\ln\varphi_{0}}{\ln\left(r_{\min,\;0}/r_{\max,\;0}\right)}\\ D_{T0}=1+\frac{\ln{\bar{\tau }}_{0}}{\ln\left(\frac{D_{f0}-1}{\sqrt{D_{f0}}}\sqrt{\frac{1-\varphi_{0}}{4\varphi_{0}}\frac{\pi }{2-D_{f0}}}\frac{r_{\max,\;0}}{r_{\min,\;0}}\right)}\\ {\bar{\tau }}_{0}=\frac{1}{2}\left[\begin{array}{c} 1+\frac{1}{2}\sqrt{1-{\left(r_{\min,\;0}/r_{\max,\;0}\right)}^{2-D_{f0}}}\\ +\frac{\sqrt{{\left[1-\sqrt{1-{\left(r_{\min,\;0}/r_{\max,\;0}\right)}^{2-D_{f0}}}\right]}^{2}+\frac{1}{4}\left[1-{\left(r_{\min,\;0}/r_{\max,\;0}\right)}^{2-D_{f0}}\right]}}{1-\sqrt{1-{\left(r_{\min,\;0}/r_{\max,\;0}\right)}^{2-D_{f0}}}} \end{array}\right] \end{array}\right..$$

In the above two equations, parameter $D_{T}0$ denotes the initial tortuosity fractal dimension (dimensionless), parameter $D_{f}0$ denotes the initial pore fractal dimension (dimensionless), $\sigma_{v}$ denotes the tangential momentum accommodation coefficient (dimensionless), ξ denotes the gas mean free path (μm), ${\bar{\tau }}_{0}$ denotes the initial average tortuosity(dimensionless).

Equation (8) takes the nanoscale effect (gas slippage) into account. If the nanoscale effect is ignored, Equation (8) can be rewritten as

(10) $$K_{0}=\frac{2^{D_{T0}}\times \pi \times D_{f0}\times r_{\max,\;0}^{D_{f0}-D_{T0}-1}}{16\times {\left(\frac{\pi \times D_{f0}}{2-D_{f0}}\times \frac{1-\varphi_{0}}{\varphi_{0}}\right)}^{\frac{D_{T0}+1}{2}}}\times \frac{r_{\max,\;0}^{3+D_{T0}-D_{f0}}-r_{\min,\;0}^{3+D_{T0}-D_{f0}}}{3+D_{T0}-D_{f0}},$$

which is identical to the model derived by Xu et al. [71]. Lei et al. [14,34,57] suggested the porosity φ after deformation could be

(11) $$\left\{\begin{array}{c} \varphi ={\left(\frac{r_{\min}}{r_{\max}}\right)}^{2-D_{f}}\\ D_{f}=2+\frac{\left(D_{f0}-2\right)r_{\max,\;0}}{\left(3-D_{f0}\right)r_{\max}+\left(D_{f0}-2\right)r_{\max,\;0}} \end{array}\right.,$$

where parameter $D_{f}$ denotes the pore fractal dimension of tight porous media (nanoporous media) after deformation (dimensionless).

Then, the permeability of nanoporous *K* media during the pressure loading process is

(12) $$K=\frac{2^{D_{T}}\times \pi \times D_{f}\times r_{\max}^{D_{f}-D_{T}-1}}{16\times {\left(\frac{\pi \times D_{f}}{2-D_{f}}\times \frac{1-\varphi_{0}}{\varphi_{0}}\right)}^{\frac{D_{T}+1}{2}}}\left(\begin{array}{c} \frac{r_{\max}^{3+D_{T}-D_{f}}-r_{\min}^{3+D_{T}-D_{f}}}{3+D_{T}-D_{f}}+\\ 4\frac{2-\sigma_{v}}{\sigma_{v}}\frac{\xi }{1-b_{0}\xi }\frac{r_{\max}^{2+D_{T}-D_{f}}-r_{\min}^{2+D_{T}-D_{f}}}{2+D_{T}-D_{f}} \end{array}\right),$$

where *D*${}_{T}$ in Equation (12) denotes the tortuosity fractal dimension of tight porous media after deformation (dimensionless) which can be determined by Equation (A8). Then, by combining Equations (8) and (12), the normalized permeability *K*${}_{d}$ during the pressure loading process is

(13) $$K_{d}=K/K_{0},$$

where subscript “d” means the normalized value.

Physically speaking, during the pressure unloading process (i.e., effective stress-reducing process), the contact radius of particles will decrease as effective stress decreases, while the arrangement of particles can never recover. Assuming the effective stress $\sigma_{\mathrm{xp},}\;\max$ (subscript “xp” means the value in pressure unloading process) and contact angle $\theta_{\mathrm{xp}}$ correspond to the onset of the pressure unloading process. Because of Hertz deformation theory, the following equation for contact radius *a* is given by

(14) $$a=\sqrt{R^{2}-b^{2}}=\sqrt{R^{2}-2F_{\mathrm{xp}}/\left(\pi \sigma_{\mathrm{xp}}\right)}\;\left(\sigma_{0}\leq \sigma_{\mathrm{xp}}\leq \sigma_{\mathrm{xp},}\;\max\right).$$

By combining Equations (7) and (14), the pore radius during the pressure unloading process is

(15) $$\left\{\begin{array}{c} r_{\mathrm{xp},\;\max\;}=R_{\max}\sqrt{\frac{4b_{\max}^{2}}{\pi R_{\max}^{2}}\sin\theta_{1}^{\prime }-\frac{4a_{\max}b_{\max}}{\pi R_{\max}^{2}}-\left(1-\frac{4}{\pi }\arctan\frac{a_{\max}}{b_{\max}}\right)}\\ r_{\mathrm{xp},\;\min}=R_{\min}\sqrt{\frac{4b_{\min}^{2}}{\pi R_{\min}^{2}}\sin\theta_{1}^{\prime }-\frac{4a_{\min}b_{\min}}{\pi R_{\min}^{2}}-\left(1-\frac{4}{\pi }\arctan\frac{a_{\min}}{b_{\min}}\right)} \end{array}\right.,$$

where the contact angle $\theta_{1}^{\prime }$ during the pressure unloading process (unit: rad) can be determined as [33]

(16) $$\theta_{1}^{\prime }=\theta_{1}\left[1-\gamma {\left(\frac{\sigma_{\mathrm{xp},\;\max}-\sigma_{\mathrm{xp}}}{\sigma_{\mathrm{xp},\;\max}-\sigma_{0}}\right)}^{\lambda }\right].$$

In the above equation, parameter γ (dimensionless) denotes the parameter reflecting the change extent of contact angle $\theta_{1}^{\prime }$. Mathematically speaking, it determines the interval of variation of $\theta_{1}^{\prime }$. In addition, parameter λ (dimensionless) denotes the parameter reflecting the change rate of contact angle $\theta_{1}^{\prime }$. Equation (16) reveals that, during the pressure unloading process ($\sigma_{\mathrm{xp}}$ changes from $\sigma_{\mathrm{xp},\;\max}$ to $\sigma_{0}$), the contact angle $\theta_{1}^{\prime }$ changes from $\theta_{1}{|}_{\sigma_{\mathrm{xp},\;\max}}$ to $\theta_{1}{|}_{\sigma_{0}}\times \left(1-\gamma \right)$. Mathematically, we have $0\leq \gamma {\left(\frac{\sigma_{\mathrm{xp},\;\max}-\sigma_{\mathrm{xp}}}{\sigma_{\mathrm{xp},\;\max}-\sigma_{0}}\right)}^{\lambda }\leq 1$. As a result, parameter $\theta_{1}^{\prime }$ is smaller than $\theta_{1}$, which implies that even if the effective stress decreases to its original value, the contact angle can never return to its original state (i.e., the pore structure can never recover). If the effective stress decreases to the initial value $\sigma_{0}$, the contact angle can be determined as $\theta_{1}^{\prime }=\theta_{1}\left(1-\gamma \right)$. So, parameter γ denotes the decreasing extent of contact angle after effective stress decreases to the initial value. Furthermore, the parameter λ in Equation (16) represents the speed of contact angle recovery. In other words, it determines how fast the contact angle recovers during the pressure unloading process.

Based on Equations (15), (16), (A8) and (A10), the permeability during the pressure unloading process $K_{\mathrm{xp}}$ is

(17) $$K_{xp}=\frac{2^{D_{T,\;\mathrm{xp}}}\times \pi \times D_{f,\;\mathrm{xp}}\times r_{\mathrm{xp},\;\max}^{D_{f,\;\mathrm{xp}}-D_{T,\;\mathrm{xp}}-1}}{16\times {\left(\frac{\pi \times D_{f,\;\mathrm{xp}}}{2-D_{f,\;\mathrm{xp}}}\right)}^{\frac{D_{T,\;\mathrm{xp}}+1}{2}}{\left(\frac{r_{\mathrm{xp},\;\max}^{2-D_{f,\;\mathrm{xp}}}}{r_{\mathrm{xp},\;\min}^{2-D_{f,\;\mathrm{xp}}}}-1\right)}^{\frac{D_{T,\;\mathrm{xp}}+1}{2}}}\left(\begin{array}{c} \frac{r_{\mathrm{xp},\;\max}^{3+D_{T,\;\mathrm{xp}}-D_{f,\;\mathrm{xp}}}-r_{\mathrm{xp},\;\min}^{3+D_{T,\;\mathrm{xp}}-D_{f,\;\mathrm{xp}}}}{3+D_{T,\;\mathrm{xp}}-D_{f,\;\mathrm{xp}}}+\\ 4\frac{2-\sigma_{v}}{\sigma_{v}}\frac{\xi }{1-b_{0}\xi }\frac{r_{\mathrm{xp},\;\max}^{2+D_{T,\;\mathrm{xp}}-D_{f,\;\mathrm{xp}}}-r_{\mathrm{xp},\;\min}^{2+D_{T,\;\mathrm{xp}}-D_{f,\;\mathrm{xp}}}}{2+D_{T,\;\mathrm{xp}}-D_{f,\;\mathrm{xp}}} \end{array}\right),$$

where

(18) $$\left\{\begin{array}{c} D_{f,\;\mathrm{xp}}=2+\frac{\left(D_{f0}-2\right)r_{\max,}\;0}{\left(3-D_{f0}\right)r_{\mathrm{xp},\;\max}+\left(D_{f0}-2\right)r_{\max,}\;0}\\ \varphi_{\mathrm{xp}}={\left(\frac{r_{\mathrm{xp},\;\min}}{r_{\mathrm{xp},\;\max}}\right)}^{2-D_{f,\;\mathrm{xp}}}\\ D_{T,\;\mathrm{xp}}=1+\frac{\ln{\bar{\tau }}_{\mathrm{xp}}}{\ln\left(\frac{D_{f,\;\mathrm{xp}}-1}{\sqrt{D_{f,\;\mathrm{xp}}}}\sqrt{\frac{1-\varphi_{\mathrm{xp}}}{4\varphi_{\mathrm{xp}}}\frac{\pi }{2-D_{f,\;\mathrm{xp}}}}\frac{r_{\mathrm{xp},}\;\max}{r_{\mathrm{xp},}\;\min}\right)}\\ {\bar{\tau }}_{\mathrm{xp}}=\frac{1}{2}\left[\begin{array}{c} 1+\frac{1}{2}\sqrt{1-{\left(r_{\mathrm{xp},\;\min}/r_{\mathrm{xp},\;\max}\right)}^{2-D_{f,\;\mathrm{xp}}}}\\ +\frac{\sqrt{{\left[1-\sqrt{1-{\left(r_{\mathrm{xp},\;\min}/r_{\mathrm{xp},\;\max}\right)}^{2-D_{f,\;\mathrm{xp}}}}\right]}^{2}+\frac{1}{4}\left[1-{\left(r_{\mathrm{xp},\;\min}/r_{\mathrm{xp},\;\max}\right)}^{2-D_{f,\;\mathrm{xp}}}\right]}}{1-\sqrt{1-{\left(r_{\mathrm{xp},\;\min}/r_{\mathrm{xp},\;\max}\right)}^{2-D_{f,\;\mathrm{xp}}}}} \end{array}\right] \end{array}\right.,$$

where $\varphi_{\mathrm{xp}}$ is stress-dependent porosity during the pressure unloading process. By combining Equations (8) and (17), the normalized permeability during the pressure unloading process is

(19) $$K_{d}=K_{\mathrm{xp}}/K_{0}.$$

#### 2.2.3. Workflow of Permeability Hysteresis Determination

**Figure 3:**
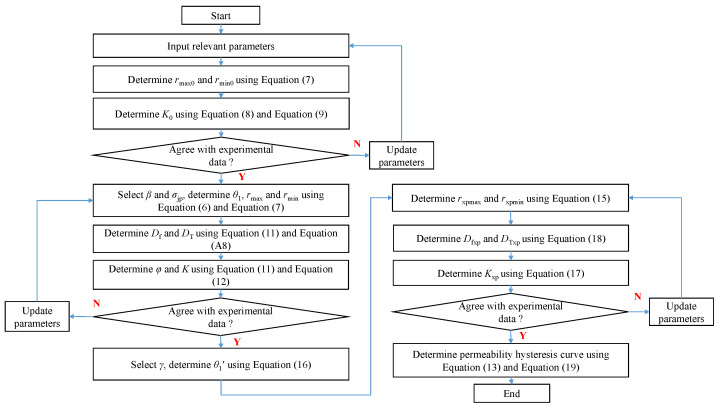
Solution procedure for the derived model.

Figure 3 presents the determination process of the hysteretic pressure-sensitive permeability of nanohybrids by using the proposed model. The suggested methodology workflow is summarized:

Step 1: Input relevant parameters (e.g., $R_{\max}$, $R_{\min}$, $\varphi_{0}$, $\sigma_{v}$, $b_{0}$, $p_{\mathrm{av}}$, *d*, $k_{B}$, *T*, $\alpha_{0}$, $\alpha_{1}$, τ). In this paper, the average pressure between the inlet and outlet of the nanohybrid $p_{\mathrm{av}}=$ 2 MPa, the elastic modulus E= 34.9 GPa, the Poisson’s ratio ν= 0.25, the temperature T= 298.15 K, and the Boltzmann constant $k_{B}=$ 1.3806505 ×$10^{-23}$ J/K. In addition, the parameter $\sigma_{v}=1$, $b_{0}=-1$, d= 3.64 ×$10^{-10}$ m, the rarefaction coefficient $\alpha_{0}=$ 1.19, parameter $\alpha_{1}=$ 4, and τ= 0.4 [22]. During the modeling, $\varphi_{0}$ should be identical to the experimental data. Determine the initial permeability $K_{0}$ using Equations (8) and (9). If the predicted $K_{0}$ is inconsistent with the measured value, update the parameters $R_{\max}$ and $R_{\min}$ until the predicted $K_{0}$ is consistent with the measured initial permeability.

Step 2: Select parameters β, Π, $\sigma_{0}$, and $\sigma_{\mathrm{xp},\;\max}$. Determine the parameters $\theta_{1}$, $r_{\max}$, and $r_{\min}$ of nanohybrid during the pressure loading process using Equations (6) and (7). Then, the permeability *K* during the pressure loading process can be calculated using Equations (A8), (11) and (12). During the modeling in this stage, $\sigma_{0}$ and $\sigma_{\mathrm{xp},\;\max}$ should be assigned based on the experimental conditions. In addition, parameters ω, β, and Π will be updated to make sure the predicted permeability curves during the pressure loading process are consistent with the measured results.

Step 3: Select the parameters γ and λ, and calculate the parameter $\theta_{1}^{\prime }$ using Equation (16). Then, the parameters $r_{\mathrm{xp},\;\max}$ and $r_{\mathrm{xp},\;\min}$ can be determined with Equation (15). Finally, the permeability $K_{\mathrm{xp}}$ during the pressure unloading process can be calculated using Equations (17) and (18). Mathematically speaking, during the modeling in this stage, parameters γ and λ will be updated to make sure the predicted permeability curves under the pressure unloading process are consistent with the measured results.

Step 4: By combining Equations (11) and (18), φ and $\varphi_{\mathrm{xp}}$ can be determined. Then, determine the permeability hysteresis curve according to Equations (13) and (19).

## 3. Results

### 3.1. Validation with Experimental Results

**Figure 4:**
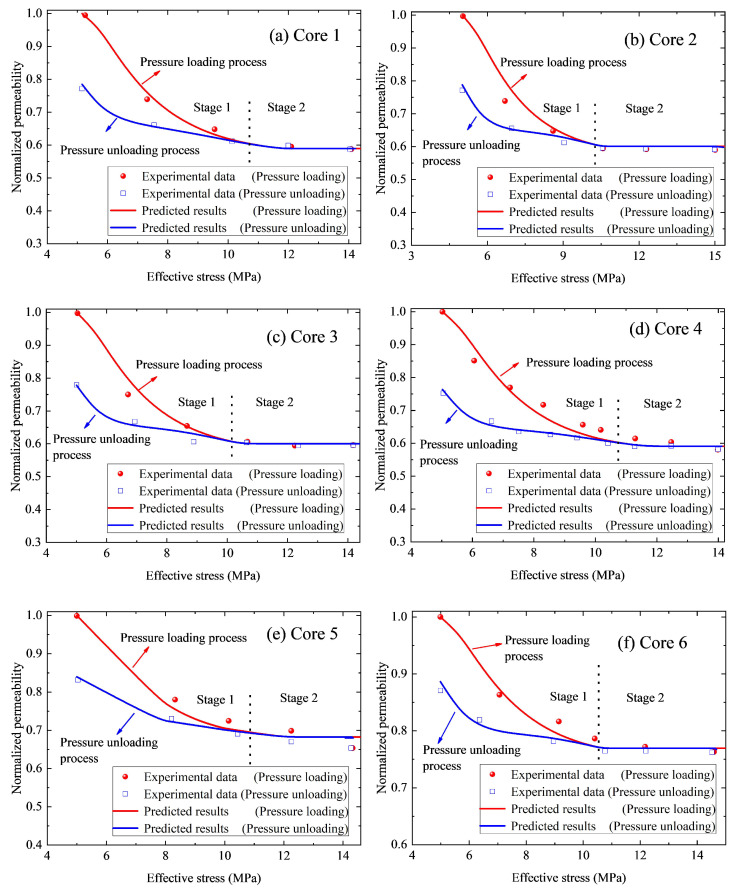
Experimental permeability data versus predicted permeability: (**a**) Core 1. (**b**) Core 2. (**c**) Core 3. (**d**) Core 4. (**e**) Core 5. (**f**) Core 6.

To verify the derived hysteretic pressure-sensitive permeability, the experimental data in Figure 1 are used. The predictions from the derived model are compared in Figure 4. To ensure the initial petrophysical properties ($\varphi_{0}$ and $K_{0}$) of the simulated nanohybrids during the simulation are identical to those of experimental samples, the relevant parameters are assigned and summarized in Table 2.

It should be noted that, besides the parameters listed in Table 2, other parameters applied in the modeling are the same as those stated in Step 1 of the workflow. Results depicted in Table 2 suggest that the calculated initial permeability $K_{0}$ of each nanoporous media is consistent with that of the experimental data, which, to some extent, reveals that the parameters assigned in the calculations are suitable and reasonable. As plotted in Figure 4, the calculations (e.g., the normalized permeability) of our derived model fit well with the experimental results. It can be understood that, for a given core sample, once the relevant parameters in the proposed model are determined accurately by fitting the available experimental data, this derived model can be used to predict permeability under other effective stresses.

**Figure 5:**
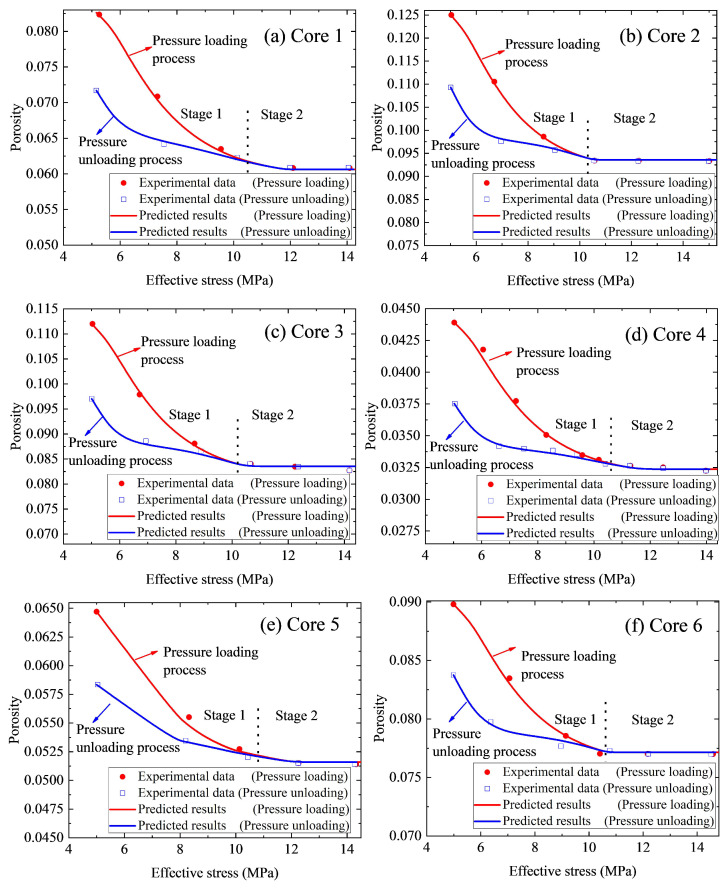
Experimental porosity data versus predicted porosity: (**a**) Core 1. (**b**) Core 2. (**c**) Core 3. (**d**) Core 4. (**e**) Core 5. (**f**) Core 6.

To further validate our derived model, Figure 5 compares the experimental porosity data with the predicted porosity. We can also see that the predicted porosity is also consistent with the experimental results, which reveals the derived model significantly coordinates with the experimental data. Moreover, it can be seen from Figure 4 and Figure 5 that, compared with the predicted permeability, the predicted porosity is more consistent with the experimental results. Specifically, the maximum relative error between the calculated permeability and the measured results is 6.08%. However, the calculated porosity is more in agreement with the experimental results with the maximum relative error of 0.5%. It is because that, compared to porosity, permeability is more sensitive to effective stress. Thus, under certain effective stress, the variation in permeability is greater than that in porosity. In general, during the permeability tests, both the permeability and porosity under differ-ent effective stresses can be measured. However, compared to porosity, permeability is more sensitive to the test condition (e.g., the experiment speed and the confining pressure), thus, the permeability test data is more scattered than the measured porosity data. As a result, the calculated porosity is more consistent with the experimental results. Moreover, Figure 4 depicts that the permeability curve of the pressure loading process or pressure unloading process can be divided into two stages: stage 1 (e.g., effective stress σ is smaller than $\sigma_{0}\Pi$) and stage 2 (e.g., effective stress σ is larger than $\sigma_{0}\Pi$). At stage 1, the difference between permeabilities for the pressure loading process and the pressure unloading process is obvious. However, during stage 2, the two curves almost coincided, and the discrepancy between these two curves is within the precision interval and can be ignored. In general, during stage 2, structural deformation almost does not vary and solid material deformation is the dominant deformation mechanism. Thus, it is reasonable and safe to infer that the structural deformation of nanoporous media is one of the principal reasons for permeability hysteresis.

### 3.2. Sensitivity Analysis

**Figure 6:**
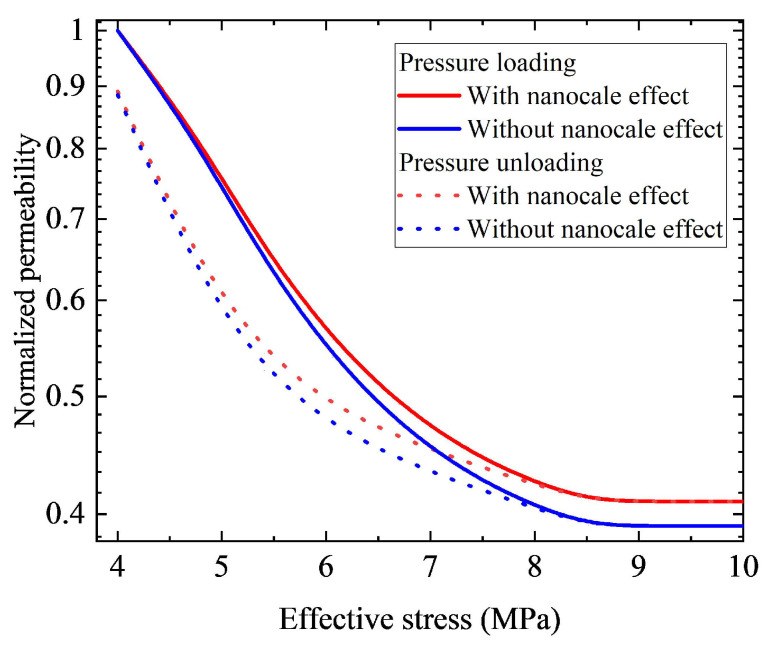
Permeability hysteresis curves with and without nanoscale effect.

After validation by these experimental results, this proposed model is applied for sensitivity analysis. Figure 6 presents permeability hysteresis curves with and without the nanoscale effect (gas slippage). During the simulation, when the nanoscale effect is taken into account, the initial maximum pore radius $r_{\max,\;0}$ is assigned as 21.6 nm, and the initial minimum pore radius $r_{\min,\;0}$ is assigned as 0.2 nm, and other parameters assigned are identical to those applied in Core 6 for the validation. However, when the nanoscale effect is ignored, parameter ξ is assigned as 0. It can be observed from Figure 6 that, under a given effective stress, the normalized permeability with the nanoscale effect (gas slippage) during the pressure loading/unloading process is larger than that without the nanoscale effect. That is to say, if the gas slippage effect is ignored in nanopores, the permeability of the nanohybrid reduces dramatically. It is owing to that, under the same condition, the gas slippage effect will increase the flow velocity of the fluid, increasing the permeability of nanohybrids. With all mentioned above, the impact of nanoscale effects on permeability is considerable and should not be neglected in nanohybrid.

**Figure 7:**
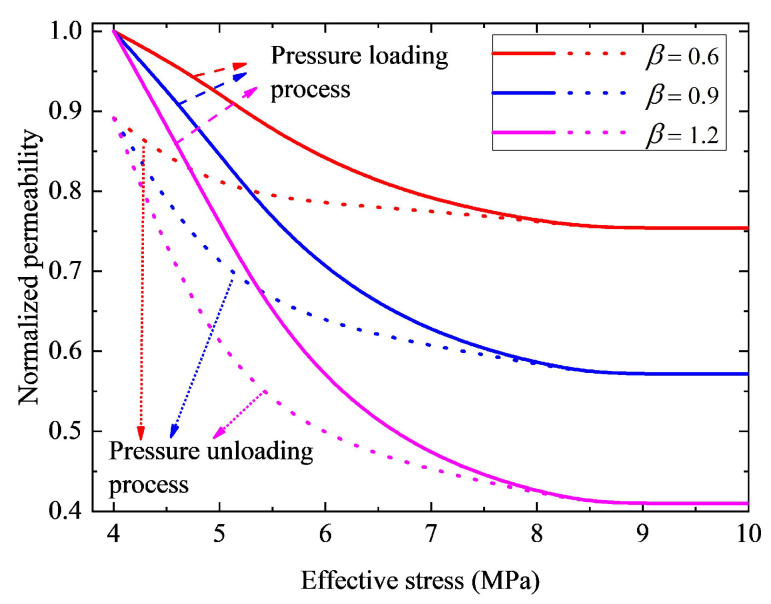
Effect of parameter β on permeability hysteresis curve. The solid lines indicate the pressure loading process curves, and the dashed lines represent curves of the pressure unloading process.

Figure 7 presents the influence of parameter β-value on normalized permeability hysteresis curves. During the simulation, the parameters assigned are identical to those applied in Figure 6. The values of these parameters assigned are the same hereinafter. It can be observed from Figure 7 that the normalized permeability decreases as the parameter β-value increases. The probable reason is that a larger parameter β-value means a faster change rate of contact angle and a stronger structural deformation of nanoporous media, leading to a narrower pore radius and a lower permeability. Moreover, as the function of petrophysical properties of reservoirs, the parameter β-value varies with different nanohybrids. Therefore, to reduce model uncertainty, the parameter β should be evaluated for every nanohybrid type.

**Figure 8:**
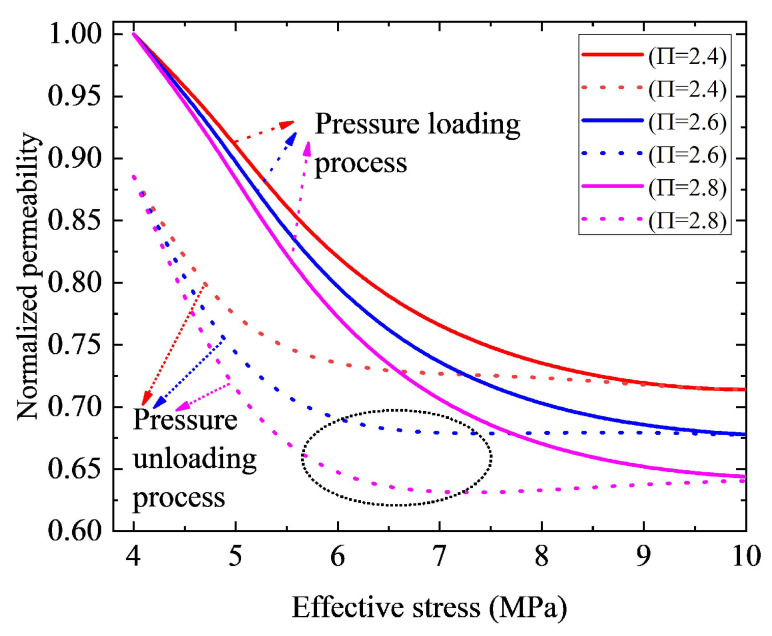
Effect of parameter Π on permeability hysteresis curve. The solid lines indicate the pressure loading process curves, and the dashed lines represent curves of the pressure unloading process.

By fitting the derived model with the experimental data, the recommended value for parameter Π is approximately 2 [33]. However, as parameter Π is related to the mineralogical composition and pore structures of nanohybrids, its value varies with different rocks. Figure 8 presents the influence of parameter Π value on normalized permeability curves. As plotted in Figure 8, a larger parameter Π corresponds to lower permeability. As a larger parameter Π means a stronger structural deformation, permeability decreases as parameter Π increases. In other words, permeability reduction due to the structural deformation increases as parameter Π increases. However, it should be noted that the permeability of nanoporous media after the stress returning to the original state (4 MPa) is independent of parameter Π, which illustrates that parameter Π affects the compression process. However, the final state of pores after the stress returning to the original state is not influenced by parameter Π. Furthermore, we can see from Figure 8 that, in this case, the permeability curve during the pressure unloading process shows a complicated shape, which is not monotonic. When the parameter Π is smaller than a certain value (Π≤ 2.6 in this case), permeability increases as effective stress decreases. However, when the parameter Π increases up to this certain value (Π≥ 2.6 in this case), the permeability curve during the pressure unloading process is not monotonic. Still, it presents a complicated shape (e.g., the permeability curve presents a V-shape in the black circle area in Figure 8). The primary reasons will be explained as follows.

**Figure 9:**
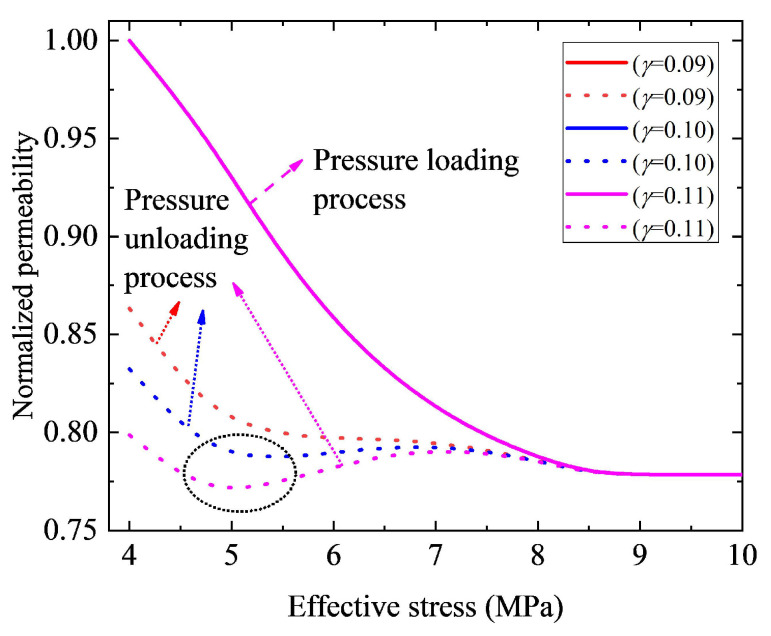
Effect of parameter γ on permeability hysteresis curve. The solid lines indicate the pressure loading process curves, and the dashed lines represent curves of the pressure unloading process.

**Figure 10:**
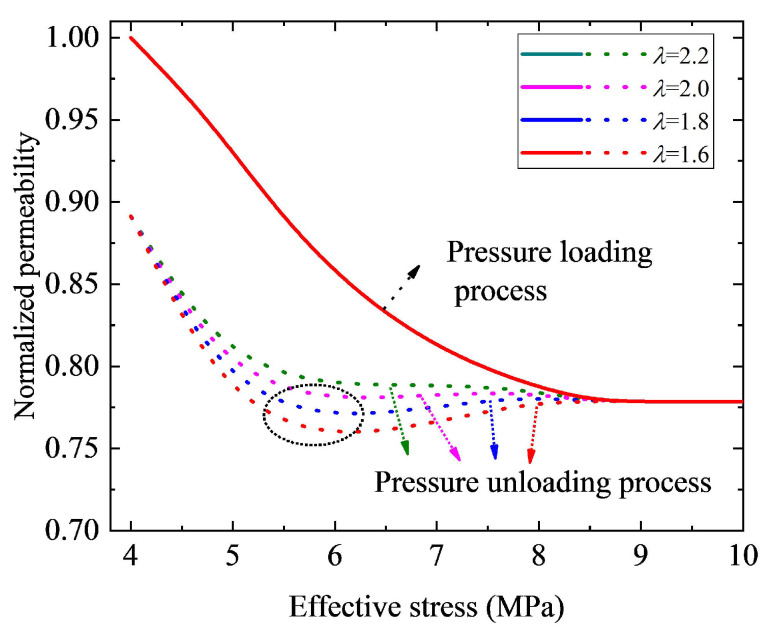
Effect of parameter λ on permeability hysteresis curve. The solid lines indicate the pressure loading process curves, and the dashed lines represent curves of the pressure unloading process.

Equation (16) indicates that the contact angle during the pressure unloading process is related to parameters γ and λ. In this study, we will study the effect of these two parameters on hysteretic pressure-sensitive permeability. Physically speaking, parameters γ and λ reflect the variation of pore structure during the unloading process. As a result, the permeability curve during loading pressure will not be affected by these two parameters. Figure 9 displays the curves of permeability versus effective stress with different values of parameter γ. The results in Figure 9 show that, for a small value of parameter γ, the permeability curve is monotonic during pressure recovery. Specifically, permeability increases as effective stress decreases. However, when the value of parameter γ increases up to a certain extent (γ≥ 0.10 in this case), the permeability curve during the pressure unloading process is not monotonic, but presents a complicated shape (e.g., the permeability curve presents a V-shape in the black circle area in Figure 9). In addition, a normalized permeability loss increases with the increase of parameter γ. It is chiefly because of that, during the pressure unloading process, when the effective stress returns to $\sigma_{0}$ (the original state), the contact angle will change from $\theta_{1}{|}_{\sigma_{0}}$ to $\theta_{1}{|}_{\sigma_{0}}\times \left(1-\gamma \right)$ with the loss of $\theta_{1}{|}_{\sigma_{0}}\times \left(\gamma \right)$. Thus, a smaller value of γ means a smaller final loss of contact angle, leading to a smaller permeability loss. On the contrary, a larger value of γ corresponds to a larger final loss of contact angle, resulting in a smaller permeability value. As depicted in Figure 10, during the pressure loading process, permeability is not influenced by parameter λ. However, it is shown that, for a small value of parameter λ, the permeability curve during the pressure unloading process shows a complicated shape. However, when the value of parameter λ increases up to a certain extent (λ≥ 2.2 in this case), permeability increases as parameter λ increases with given effective stress during the pressure unloading process. Furthermore, we can see that normalized permeability loss is independent of λ. The main reason for this is that, parameter λ affects the speed of contact angle recovery instead of the final loss of contact angle during the pressure unloading process. As a result, it does not affect the pressure loading process. In addition, during the pressure unloading process, a larger value of λ means a fast speed of contact angle recovery and a larger contact angle value, leading to a larger permeability value. However, as parameter λ does not affect the final loss of contact angle, it has no effect on the final loss of permeability of nanohybrids.

By combining Equations (6) and (16), with the condition $\frac{\sigma }{\sigma_{0}}\leq \Pi$, we have

(20) $$\theta_{1}^{\prime }=\left(\frac{\pi }{3}+\frac{\pi }{6}{\left(\frac{\sigma_{0}}{\sigma }\right)}^{\frac{\sigma }{\Pi \sigma_{0}}\beta }\right)\left[1-\gamma {\left(\frac{\sigma_{\mathrm{xp},\;\max}-\sigma }{\sigma_{\mathrm{xp},\;\max}-\sigma_{0}}\right)}^{\lambda }\right]=f\left(\sigma \right)\times g\left(\sigma \right).$$

Mathematically, f(σ) monotonically decreases with the increasing effective stress σ, which ranges from $\frac{\pi }{2}$ to $\left(\frac{\pi }{3}+\frac{\pi }{6\Pi^{\beta }}\right)$, when effective stress increases from $\sigma_{0}$ to $\sigma_{0}\Pi$. However, g(σ) monotonically increases with the increasing effective stress σ, which ranges from (1−γ) to $\left[1-\gamma {\left(\frac{\sigma_{\mathrm{xp},\;\max}-\Pi \sigma_{0}}{\sigma_{\mathrm{xp},\;\max}-\sigma_{0}}\right)}^{\lambda }\right]$, when effective stress increases from $\sigma_{0}$ to $\sigma_{0}\Pi$. As the contact angle $\theta_{1}^{\prime }$, during the pressure unloading process, is affected by the comprehensive impact of f(σ) and g(σ), its monotonicity is complicated. Mathematically speaking, if the parameter $\theta_{1}^{\prime }$ is monotonically increasing as the effective stress changes from $\sigma_{\mathrm{xp},\;\max}$ to $\sigma_{0}$, the corresponding permeability will monotonically increase during this process. However, it is found that, during the pressure unloading process (i.e., the effective stress decreases from $\sigma_{\mathrm{xp},\;\max}$ to $\sigma_{0}$), the variations are not monotone, parameter $\theta_{1}^{\prime }$ will firstly decrease and then increase, leading to permeability firstly decreases and then increases as the effective stress changes from $\sigma_{\mathrm{xp},\;\max}$ to $\sigma_{0}$. Thus, parameter $\theta_{1}^{\prime }$ will be comprehensively affected by the relevant parameters (e.g., β, Π, γ, and λ). For example, when other parameters are given, for a small parameter γ, contact angle $\theta_{1}^{\prime }$ is a monotonically decreasing function of effective stress. However, when parameter γ increases up to a certain value, the monotonicity of the contact angle $\theta_{1}^{\prime }$ changes. Specifically, for a larger γ, when the effective stress decreases from $\sigma_{\mathrm{xp},\;\max}$ to $\sigma_{0}$, parameter $\theta_{1}^{\prime }$ will firstly decrease then increase, resulting in permeability firstly decreasing and then increasing during this process. Thus, permeability curves during the pressure unloading process present complicated shapes, as shown in Figure 8, Figure 9 and Figure 10. To improve the model accuracy of modeling permeability, relevant parameters (e.g., β, Π, γ, and λ) should be carefully checked for every type of tight porous media.

## 4. Discussion

As an attempt to characterize the permeability hysteresis effect of nanoporous media, the derived model can provide theoretical foundations for quantifying the deformation of nanohybrids. Compared with the experimental tests, which are commonly expensive, time-consuming, and vulnerable to extraneous variables, the derived model can reveal the mechanism of the deformation of nanohybrids and explain the results. Moreover, for the coupled flow deformation problem stated here, the numerical models (e.g., the Lattice Boltzmann method and the Finite Element method) need accurate pore structure information of the porous materials [72,73,74]. For example, if the detailed pore structure information is unavailable, the permeability of porous materials cannot be obtained accuracy by numerical simulation. In addition, the numerical models require large computer memory and storage space to reduce the numerical errors caused by the poor discretization schemes (coarse meshing) and low space resolution of the pore structure, and are vulnerable to numerical dispersion. Moreover, numerical modeling is with great uncertainty caused by the complex nature of nanoporous media. Compared with the numerical models, the derived model is robust and free from discretization errors. Specifically, the derived model can alleviate the computational burden when modeling the deformation of nanohybrids.

However, it should be noted that the derived model needs to be extended to address its limitations. For example, the derived model ignores the effect of water film on the permeability of nanohybrids. Zhang et al. [22] suggested that the absorption of water film on the pore surface would affect fluid flow in nanohybrids significantly. Thus, for actual nanohybrids, the effect of water film on nanohybrids permeability can not be ignored. Moreover, this derived model is a capillary bundle model which ignores the interconnection between pores. In general, this capillary tube-based model without the interplay between pores may underestimate the flow resistance and thus overestimate the permeability of the nanoporous media. In addition, this study is for intact rocks, which ignores the deformation of joints and fractures. Moreover, this model is suitable for circular nanopores. However, for fluid flow through nanopores, the effect of the actual irregular shape of nanopores on flow behavior is significant [75]. Furhtermore, this study is limited to the fully elastic deformation of tight porous media. With the increase of effective stress, elastoplastic deformation and fully plastic deformation will occur, which causes the problem more complicated. In our further work, elastoplastic deformation and fully plastic deformation of nanoporous media due to the effective stress change will be taken into account to make more accurate predictions. In this paper, the pores in the nanohybrids are assumed to be framed by the cubic packing of the same particles with neglecting the polydispersity of particle packing. In addition, the fluid velocity is assumed to be approximately symmetrically distributed with respect to the tube axis, however, for fluid flow in random and disordered pore space, the velocity distribution will be extremely complicated. Thus, the imposed boundary conditions are a relatively simple representation of the flow in nanohybrids.

Furthermore, the effect of stress relaxation on the mechanical properties of nanohybrids (e.g., elastic modulus and Poisson’s ratio) are ignored in this study. In general, nanohybrids mechanical properties will be affected by effective stress, and the behavior of the nanohybrids load-bearing structure is stress-dependent. Physically speaking, nanohybrids load-bearing structure parameters will alter the area over which the effective stress is distributed. Hence, nanohybrids mechanical properties would be affected by effective stress. As a result, it is critical to study the influence of effective stress on the mechanical properties of nanohybrids. Consequently, the variation in pore structure during pressure loading and unloading processes is complex. This model should be extended in the future.

## 5. Conclusions

In this study, a new fractal-based analytical model is derived for characterizing the permeability hysteresis effect of nanohybrids during the process of pressure change. The model predictions agree with the experimental results and reveal that the permeability hysteresis effect is nontrivial for nanohybrids. The proposed model indicates that the hysteretic pressure-sensitive permeability of nanoporous media is affected by effective stress, nanoscale effect (gas slippage), pore microstructure parameters, grain quadrilateral angle, and the loss rate of grain quadrilateral angle. Compared with the conventional porous media, nanohybrid permeability increases dramatically due to the gas slippage effect. During the pressure unloading process, permeability is not a monotonous function and presents complicated shapes, as affected by the comprehensive impact of multiple influential parameters. As relevant influential parameters are functions of mineralogical composition and pore structures of nanohybrids, these parameters should be carefully checked for different types of nanoporous media to improve the model accuracy and reduce model uncertainty.

To the best of the authors’ knowledge, it is the very first work on hysteretic pressure-sensitive permeability of nanohybrids. The proposed model can explain, quantify and predict the hysteretic pressure-sensitive permeability of nanoporous media reasonably well. It can also be used to estimate specific parameters with inverse modeling. In addition, with this derived model, more details and information on the mechanisms that lead to the coupled flow deformation behavior in nanohybrids can be revealed and studied. However, it should be noted that some parameters in the derived model need to be determined through further study.

## Figures and Tables

**Figure A1:**
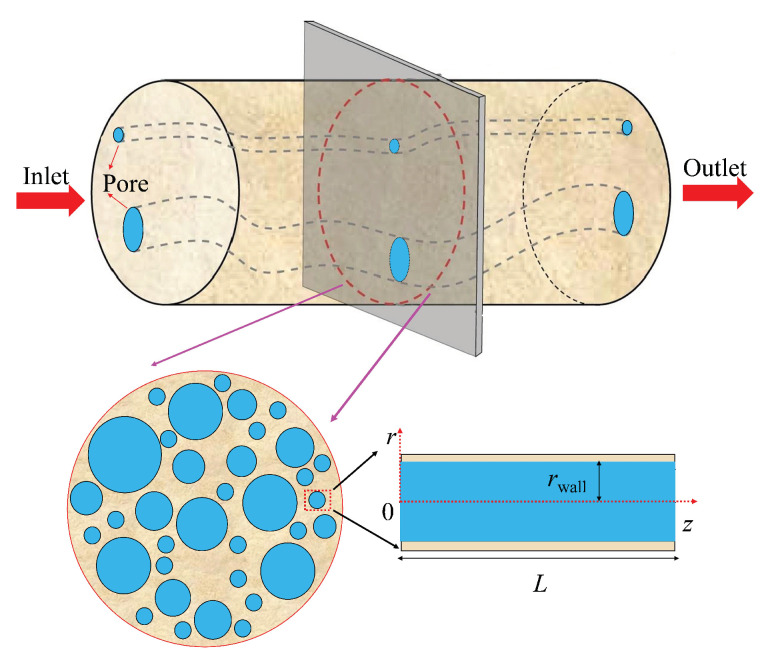
Physical conceptual model of nitrogen flow through nanoporous media.

**Table 1:** Properties of artificial sandstones (nanohybrids) used in the experiments.

No.	Diameter/mm	Length/mm	Initial Porosity/%	Initial Permeability/$10^{-3}$ μ$m^{2}$	Elastic Modulus/GPa	Poisson’s Ratio
Core-1	24.5	48.4	8.26	0.05	34.9	0.25
Core-2	24.8	52.6	12.5	0.15		
Core-3	24.7	49.7	11.2	0.12		
Core-4	25.2	43.8	4.39	0.02		
Core-5	24.9	56.2	6.47	0.04		
Core-6	25.2	51	8.98	0.06		

**Table 2:** Parameters applied in the model for the model validation.

Parameters	Core-1	Core-2	Core-3	Core-4	Core-5	Core-6
$R_{\max}$/μm	0.38	0.52	0.49	0.36	0.4	0.39
$R_{\min}$/10^−3^μm	3.15	4.59	4.18	2.75	2.64	3.63
Initial porosity/%	8.26	12.5	11.2	4.39	6.47	8.98
Predicted initial permeability/^−3^ μm^2^	0.05	0.15	0.12	0.02	0.04	0.06
ω	0.46	0.47	0.48	0.48	0.44	0.46
Π	2.3	2.1	2.1	2.32	2.4	2.14
β	0.76	0.95	0.92	0.62	0.56	0.56
γ	0.11	0.12	0.12	0.1	0.09	0.08
λ	3.6	2.8	2.9	3.3	3.6	2.6

## Data Availability

Not applicable.

## Appendix A. The Derivation of Nitrogen Flow in Nanopores

Assuming nitrogen flow through nanohybrid in the axial direction *z* under unidirectional pressure driven, as shown in Figure A1.

For nitrogen flow through a circular nanopore with pore radius $r_{\mathrm{wall}}$, based on Navier-Stokes equation with viscosity modified, the flow equation can be appropriately expressed as

(A1) $$\frac{\mu_{g}}{r}\frac{\partial }{\partial r}\left(r\frac{\partial v_{g}}{\partial r}\right)=-\frac{\Delta p}{L},$$

where $v_{g}$ is the gas velocity (cm/s), Δp is pressure gradient (MPa/m), *L* is the length of the capillary (cm), and $\mu_{g}$ is the dynamic viscosity of nitrogen (mPa·s), which is [22,62,63].

(A2) $$\left\{\begin{array}{c} \mu_{g}=\frac{\mu_{\mathrm{gi}}}{1+\alpha K_{n}}\\ \alpha =\alpha_{0}\frac{2}{\pi }\tan\left(\alpha_{1}K_{n}^{\tau }\right) \end{array}\right.,$$

where $\mu_{\mathrm{gi}}$ is the nitrogen viscosity at standard temperature and pressure state (mPa·s), which is assigned as 0.0159 mPa·s. $\alpha_{0}$ is the rarefaction coefficient when Knudsen number $K_{n}\to \infty$, $\alpha_{1}$, and τ are fitting coefficients. In this paper, these parameters are assigned as 1.19, 4, and 0.4, respectively, which are identical to those used in the literature [22]. Based on the definition of Knudsen number $K_{n}$ (dimensionless, the ratio of gas mean free path ξ with a unit of μm to the characteristic dimension), we have

(A3) $$\left\{\begin{array}{c} K_{n}=\frac{\xi }{2r}\\ \xi =\frac{k_{B}T}{\sqrt{2}\pi d^{2}p_{\mathrm{av}}} \end{array}\right.,$$

where *T* is the temperature, which is 298.15 K in this paper.

In nanopores, the gas momentum transport will be affected by gas-gas interactions and gas-solid interactions [22]. At the pore boundary (i.e., $r=r_{\mathrm{wall}}$ ), Karniadakis et al. [76] suggested that nitrogen slip velocity $v_{s}$ (cm/s) was

(A4) $${\left.v_{s}\right|}_{r=r_{\mathrm{wall}}}=-{\left.\frac{2-\sigma_{v}}{\sigma_{v}}\frac{\xi }{1-b_{0}\xi }\left(\frac{\partial v_{g}}{\partial r}\right)\right|}_{r=r_{\mathrm{wall}}},$$

where subscript “wall” denotes the pore wall. In this paper, diffuse reflection is taken into account, and $\sigma_{v}$ is assigned as 1. Parameter $b_{0}$ in Equation (A2) is the general slip coefficient, which is 0 for the first-order slip flow and −1 for the second-order slip flow. In this paper, $b_{0}$ is assigned as −1. Parameter $r_{\mathrm{wall}}$ in Equation (A2) is the pore radius.

For fluid flow in cylindrical capillary, it is assumed that the fluid velocity is approximately symmetrically distributed with respect to the tube axis (i.e., the shear stress at r=0 is zero). In addition, based on the continuity of the velocity at pore boundary (i.e., $r=r_{\mathrm{wall}}$), the boundary conditions of the model are

(A5) $$\left\{\begin{array}{c} {\left.\frac{\partial v_{g}}{\partial r}\right|}_{r=0}=0\\ {\left.v_{g}\right|}_{r=r_{\mathrm{wall}}}={\left.v_{s}\right|}_{r=r_{\mathrm{wall}}} \end{array}\right..$$

By solving Equations (A1), (A4) and (A5), we have

(A6) $$v_{g}=-\frac{\Delta p}{L}\frac{r^{2}}{4\mu_{g}}+\frac{\Delta p}{L}\frac{1}{4\mu_{g}}\left(r_{\mathrm{wall}\;}^{2}+2\frac{2-\sigma_{v}}{\sigma_{v}}\frac{\xi }{1-b_{0}\xi }r_{\mathrm{wall}\;}\right).$$

By integrating Equation (A6) over the radius of the circular nanotube, the gas flow rate $q_{g}$ (${\mathrm{cm}}^{3}/s$, subscript “g” means gas) equation is

(A7) $$q_{g}=\int_{0}^{r_{\mathrm{wall}}}v_{g}2\pi rdr=\frac{\Delta p}{L}\frac{\pi }{8\mu_{g}}\left(r_{\mathrm{wall}\;}^{4}+4\frac{2-\sigma_{v}}{\sigma_{v}}\frac{\xi }{1-b_{0}\xi }r_{\mathrm{wall}\;}^{3}\right).$$

Based on Equation (A7) and fractal theory, with the porosity φ, pore fractal dimension $D_{f}$, tortuosity fractal dimension $D_{T}$, the maximum pore radius $r_{\max}$ and the minimum pore radius $r_{\min}$, pore size distribution *f* and the total volumetric flow rate $Q_{g}$ (${\mathrm{cm}}^{3}/s$, subscript “g” means gas) through the unit cell of fractal nanohybrids is [71,77]

(A8) $$\left\{\begin{array}{c} Q_{g}={\left(\frac{r_{\max}}{r_{\min}}\right)}^{D_{f}}\int_{r_{\min}}^{r_{\max}}q_{g}fdr\\ f=D_{f}r_{\min}^{D_{f}}r^{-\left(D_{f}+1\right)}\\ L={\left(2r\right)}^{1-D_{T}}\cdot L_{0}^{D_{T}}\\ L_{0}=r_{\max}\sqrt{\frac{\pi D_{f}}{2-D_{f}}\frac{1-\varphi }{\varphi }}\\ D_{T}=1+\frac{\ln\left[\frac{1}{2}\left[1+\frac{\sqrt{1-\varphi }}{2}+\frac{\sqrt{{\left(1-\sqrt{1-\varphi }\right)}^{2}+0.25\left(1-\varphi \right)}}{1-\sqrt{1-\varphi }}\right]\right.}{\ln\left[\frac{D_{f}-1}{\sqrt{D_{f}}}\frac{r_{\max}}{r_{\min}}\sqrt{\frac{1-\varphi }{4\varphi }\frac{\pi }{2-D_{f}}}\right]} \end{array}\right..$$

By substituting Equation (A7) into Equation (A8), Equation (A8) can be rewritten as

(A9) $$Q_{g}=\frac{\pi \times \Delta p\times D_{f}\times r_{\max}^{D_{f}}}{2^{4-D_{T}}\times \mu_{g}L_{0}^{D_{T}}}\left(\frac{r_{\max}^{3+D_{T}-D_{f}}-r_{\min}^{3+D_{T}-D_{f}}}{3+D_{T}-D_{f}}+4\frac{2-\sigma_{v}}{\sigma_{v}}\frac{\xi }{1-b_{0}\xi }\frac{r_{\max}^{2+D_{T}-D_{f}}-r_{\min}^{2+D_{T}-D_{f}}}{2+D_{T}-D_{f}}\right).$$

By combining Equation (A9) and Darcy’s law, nanoporous media permeability *K* can be obtained as

(A10) $$\begin{array}{c} K=\frac{2^{D_{T}}\times \pi \times D_{f}\times r_{\max}^{D_{f}-D_{T}-1}}{16{\left(\frac{\pi D_{f}}{2-D_{f}}\frac{1-\varphi }{\varphi }\right)}^{\frac{D_{T}+1}{2}}}\\ \times \left(\frac{r_{\max}^{3+D_{T}-D_{f}}-r_{\min}^{3+D_{T}-D_{f}}}{3+D_{T}-D_{f}}+4\frac{2-\sigma_{v}}{\sigma_{v}}\frac{\xi }{1-b_{0}\xi }\frac{r_{\max}^{2+D_{T}-D_{f}}-r_{\min}^{2+D_{T}-D_{f}}}{2+D_{T}-D_{f}}\right) \end{array}.$$
